# Effects of Dietary Fucoidan Supplementation on Serum Biochemical Parameters, Small Intestinal Barrier Function, and Cecal Microbiota of Weaned Goat Kids

**DOI:** 10.3390/ani12121591

**Published:** 2022-06-20

**Authors:** Weiguang Yang, Guangzhen Guo, Jiayi Chen, Shengnan Wang, Zhenhua Gao, Zhihui Zhao, Fuquan Yin

**Affiliations:** 1 College of Coastal Agriculture Science, Guangdong Ocean University, Zhanjiang 524088, China; yangweiguang1@stu.gdou.edu.cn (W.Y.); guoguangzhen1@stu.gdou.edu.cn (G.G.); 2112004023@stu.gdou.edu.cn (J.C.); 2112004098@stu.gdou.edu.cn (S.W.); xmsgzhh@126.com (Z.G.); 2 The Key Laboratory of Animal Resources and Breed Innovation in Western Guangdong Province, Zhanjiang 524088, China

**Keywords:** fucoidan, weaned goat kids, serum biochemical parameters, small intestinal barrier, cecal microbiota

## Abstract

**Simple Summary:**

Weaning stress has negative effects on the growth and intestinal health of goat kids. The application of antibiotics can relieve weaning stress; however, their prophylactic application has motivated researchers to find alternatives to antibiotics for mitigating the weaning stress. Fucoidan is a natural polysaccharide with antioxidant and immune-modulatory properties, and it has shown beneficial effects on intestinal barrier function. In this study, dietary supplementation of fucoidan improved the small intestinal barrier function and cecal microflora in weaned goat kids. These results suggested that fucoidan can be used as a promoter of gut health in weaned goat kids.

**Abstract:**

The purpose of this study was to evaluate the effects of fucoidan supplementation on serum biochemical parameters, small intestinal barrier function, and cecal microbiota of weaned goat kids. A total of 60 2-month-old weaned castrated male goat kids (Chuanzhong black goat) were used in this 30-day experiment. The goat kids were randomly divided into four groups: a control group (CON) fed the basal diet, and three other groups supplemented with 0.1%, 0.3%, and 0.5% fucoidan in the basal diet (denoted as F1, F2, and F3 groups, respectively). The results indicated that dietary fucoidan supplementation decreased (*p* < 0.05) the activity of lactate dehydrogenase (LDH) and the content of glucose (GLU) as measured on day 15. As measured on day 30, dietary fucoidan increased (*p* < 0.05) the content of total protein (TP) and decreased the activity of aspartate aminotransferase (AST), and supplementation with 0.3% and 0.5% fucoidan decreased (*p* < 0.05) the activity of LDH. Dietary fucoidan decreased (*p* < 0.05) the content of D-lactic acid (D-LA) and the activity of diamine oxidase (DAO). Dietary fucoidan increased (*p* < 0.05) the activity of catalase (CAT) in the duodenum. Dietary 0.3% and 0.5% fucoidan enhanced (*p* < 0.05) the activity of glutathione peroxidase (GSH-Px) in the ileum, the activity of total superoxide dismutase (T-SOD) in the jejunum and ileum, and the activity of CAT in the ileum. Dietary 0.3% and 0.5% fucoidan reduced the contents of malondialdehyde (MDA) in the duodenum, jejunum, and ileum and the content of hydrogen peroxide (H_2_O_2_) in the duodenum. Dietary fucoidan increased (*p* < 0.05) the content of secretory immunoglobulin A (sIgA) in the duodenum. Supplementation of 0.3% and 0.5% fucoidan upregulated (*p* < 0.05) the gene expression of ZO-1 and claudin-1 in the duodenum, jejunum, and ileum, and dietary supplementation of 0.3% and 0.5% fucoidan upregulated (*p* < 0.05) the gene expression of occludin in the jejunum and ileum. The 16S rRNA high-throughput sequencing results showed that at the phylum level, dietary fucoidan increased (*p* < 0.05) the abundance of *Bacteroidetes* while decreasing (*p* < 0.05) the abundance of *Firmicutes*. At the genus level, dietary 0.3% and 0.5% fucoidan increased (*p* < 0.05) the abundances of *Unspecified_Ruminococcaceae*, *Unspecified_Bacteroidale, Unspecified_Clostridiales*, and *Akkermansia.* In conclusion, dietary fucoidan supplementation had positive effects on intestinal permeability, antioxidant capacity, immunity function, tight junctions, and the cecal microflora balance in weaned goat kids.

## 1. Introduction

The weaning transition is a critical period for goat kid growth. Previous studies have confirmed that weaning stress has negative consequences for growth performance, intestinal barrier function, and the balance of intestinal microflora [1,2]. Moreover, it has been suggested that weaning stress causes intestinal barrier disruption leading to diarrhea and endangering the health of the goat kids [3,4]. Thus, gut health is critical for goat kids’ growth and welfare. Formerly, antibiotics were widely used to alleviate weaning stress [5]. However, the ban on antibiotic use in feed worldwide has forced researchers to find alternatives to antibiotics. At present, increasing attention is being paid to seaweed polysaccharides due to their bioactivities in animals [6,7,8,9].

Fucoidan, a macromolecular polysaccharide rich in sulfate groups, is found in the cell walls of brown seaweed (*Pelvetia canaliculate*, *Fucus evanescens*, and *Cladosiphon okamuranus*), the mucous matrix, and several marine invertebrates [10,11]. Recent studies have shown that dietary fucoidan supplementation could maintain intestinal health by repairing the intestinal mucosa, enhancing mucosal immune function, and regulating intestinal microflora in mice and pigs [12,13,14]. In general, fucoidan has protective effects on the intestinal physical, chemical, immune, and microbial barriers [15,16]. Thus, fucoidan has been widely used as a functional feed supplement. Furthermore, dietary inclusion of fucoidan has been suggested to improve intestinal mucosal antioxidant capacity, immune function, tight junctions, and gut microbiota balance in weaning pigs [17,18,19,20]. Similar results have also been reported in chickens and fish [21,22]. However, to the best of our knowledge, reports concerning the application of fucoidan in weaned goat kids are limited. In a companion study, fucoidan dietary supplementation increased growth performance, antioxidant capacity, immune function, and intestinal morphology in weaned goat kids [23]. However, there is at present no information concerning changes in serum biochemical parameters, small intestinal barrier function, or the cecal microbial community of goat kids after fucoidan feeding. Therefore, the purpose of this study was to fill this knowledge gap.

## 2. Materials and Methods

### 2.1. Source of Fucoidan

The fucoidan used in this study was provided by Mingyue Hailin Fucoidan Biotechnology Co., Ltd. The fucoidan was a pale-yellow powder with the odor of seaweed. The fucoidan purity was determined to be 98%; the overall sugar content was 70.5%; the fucose content was 24.9%; and the sulfate ion content was 28.9%.

### 2.2. Kids, Diet and Experimental Design

The design of this study was described in an earlier paper [23]. Briefly, a total of 60 2-month-old weaned castrated male goat kids (Chuanzhong black goat) with a mean body weight of 12.5 ± 0.5 kg were used in a 30-day experiment. The control group (CON) was fed a basal diet, while the other three groups were fed the same diet supplemented with fucoidan at 0.1% (F1 group), 0.3% (F2 group), and 0.5% (F3 group). The basal diet (Table 1) was formulated to meet or exceed the nutrient requirements from the Feeding Standards of Goats, China (NY/T 861-2004). The roughage consisted of Pennisetum purpureum. The goat kids were fed twice daily (8:30 am and 5:30 pm). Water was available ad libitum. Fucoidan was manually mixed into the concentrate. The goat kids were first fed the concentrate and then the roughage.

### 2.3. Sample Collection

On days 15 and 30, blood samples were collected from the jugular vein. The blood samples were centrifuged at 3500× *g* for 10 min at 4 °C, and the serum was collected and stored at −80 °C for later analysis.

Goat kids were fasted for 12 h prior to slaughter at the end of the trial. Six goat kids of similar weight per group were selected for slaughter. To isolate the gut tissue, approximately 3 cm segments of the midpoint of the duodenum, jejunum, and ileum were collected and stored at −80 °C for gene expression analysis. The mucosal samples were collected by the method described by Wang et al. [24]. Mucosal samples were stored at −80 °C.

Finally, the cecum was opened using a sterile scalpel, and the cecal contents were separated into 15 mL tubes using sterile pipette tips. Cecal content samples were stored at −80 °C for microbiota analysis.

### 2.4. Serum Biochemical Parameters

The contents of total glyceride (TG), total cholesterol (T-CHO), glucose (GLU), total protein (TP), and the activities of alanine aminotransferase (ALT), aspartate aminotransferase (AST), and lactate dehydrogenase (LDH) were examined using commercial kits (Nanjing Jiancheng Bioengineering Institute, Nanjing, China).

### 2.5. Intestinal Permeability

The content of D-lactic acid (D-LA) and the activity of diamine oxidase (DAO) were measured with enzyme immunoassay kits (Jiangsu Meimian Industrial Co., Ltd., Nanjing, China).

### 2.6. Antioxidant Capacity of Intestinal Mucosa

The contents of malondialdehyde (MDA), hydrogen peroxide (H_2_O_2_), and the activities of total antioxidant capacity (T-AOC), total superoxide dismutase (T-SOD), catalase (CAT), and glutathione peroxidase (GSH-Px) in the intestinal mucosa were measured using commercial kits (Nanjing Jiancheng Bioengineering Institute, Nanjing, China).

### 2.7. Intestinal Mucosal Immunity

The content of secretory immunoglobulin A (sIgA) in the intestinal mucosa was estimated using an enzyme immunoassay kit (Jiangsu Meimian Industrial Co., Ltd., Nanjing, China).

### 2.8. RT-qPCR Analysis

The total RNA extraction, reverse transcription to cDNA, and real-time quantitative polymerase chain reaction analysis employed the methods described by Guo et al. [25].

The primers were designed by Primer Premier 6.0 software (Table 2). β-actin mRNA expression was used to calculate and normalize the values, and the relative mRNA expression was calculated by the 2^−^^∆∆Ct^ method.

### 2.9. DNA Extraction

The total genomic DNA from cecal content samples was extracted using the cetyltrimethylammonium bromide (CTAB) method [26]. The concentration and quality of the DNA were estimated using 1% agarose gels. Finally, samples were diluted to 1 ng/µL with sterile water.

### 2.10. 16S rRNA Gene Amplification and Illumina novaSeq Sequencing

The distinct regions (16S rRNA V3-V4) were amplified by the specific primers 341F (5′-CCTAYGGRBGCASCAG-3′) and 806R (5′-GGACTACNNGGGTATCTAAT-3′). The PCR reactions were performed with Phusion High-Fidelity PCR Master Mix^®^ (New England Biolabs) using 2 µM primers (forward and reverse) and 10 ng template DNA. Finally, purifying the pooled PCR products was performed using a Qiagen Gel Extraction Kit (Qiagen, Germany).

Sequence libraries were generated and indexed by library construction kits (Illumina, San Diego, CA, USA). The quality of the library was assessed by a Qubit 2.0 fluorometer (Thermo Scientific, Waltham, MA, USA). Then, the library was sequenced on an Illumina NovaSeq platform, and 250 bp paired-end reads were generated.

### 2.11. Statistical Analysis

The operational taxonomic units (OTUs) were clustered with 97% similarity (3% cutoff) using Uparse software [27] (v7.0.1), and chimeric sequences were identified and removed. The phylogenetic affiliation of each 16S rRNA gene sequence was analyzed by the RDP classifier against the Database software (v1.3.1) 16S rRNA database using a confidence threshold of 70%. Alpha diversity indices and beta diversity distances were calculated by QIIME software (v1.9.1). The microbial diversity of samples was estimated by alpha diversity indices (Chao1, Shannon, and Simpson indices).

All statistical analyses were performed using SPSS 26.0. One-way ANOVA and Duncan’s multi-range test were used to analyze group differences. The results were expressed as the mean ± standard error of the mean (SEM), and differences were considered significant at *p* < 0.05.

## 3. Results

### 3.1. Serum Biochemical Parameters

As shown in Table 3, on day 15 the goat kids fed diets with fucoidan had decreases in the activity of LDH and the content of GLU (*p* < 0.05). Goat kids fed the F3 diet had increased TP content compared to goat kids fed CON, F1, and F2 diets (*p* < 0.05). No significant differences were observed in the activities of ALT, AST, or the contents of TG and T-CHO among treatments.

On day 30, goat kids fed diets with fucoidan had decreased activity of AST (*p* < 0.05). Goat kids fed F2 and F3 diets had decreased activity of LDH compared to goat kids fed CON and F1 diets (*p* < 0.05). Dietary inclusion of fucoidan increased (*p* < 0.05) the content of TP compared to goat kids fed the CON diet. No significant differences were observed in the activity of ALT or the contents of TG, T-CHO, and GLU among the treatments.

### 3.2. Intestinal Permeability

**Figure 1:**
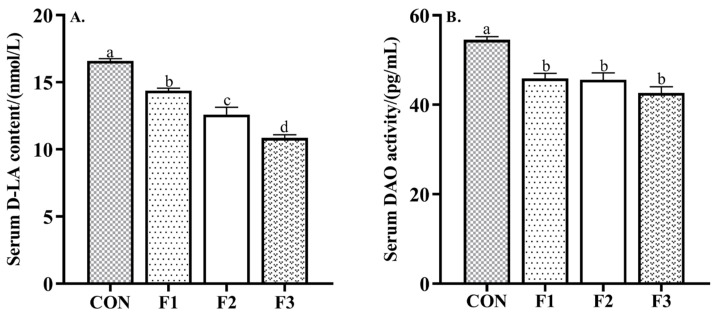
Effects of dietary fucoidan supplementation for 30 days on intestinal permeability in weaned goat kids. (**A**) Serum D-LA content; (**B**) serum DAO activity. Results are presented as mean ± SEM. ^a–d^ means significantly different (*p* < 0.05). CON, basal diet; F1, basal diet + 0.1% fucoidan; F2, basal diet + 0.3% fucoidan; F3, basal diet + 0.5% fucoidan. D-LA, D-lactic acid; DAO, diamine oxidase.

As shown in Figure 1, dietary supplementation of fucoidan decreased the content of D-LA and the activity of DAO compared to goat kids fed the CON diet (*p* < 0.05).

### 3.3. Antioxidant Capacity of Intestinal Mucosa

**Figure 2:**
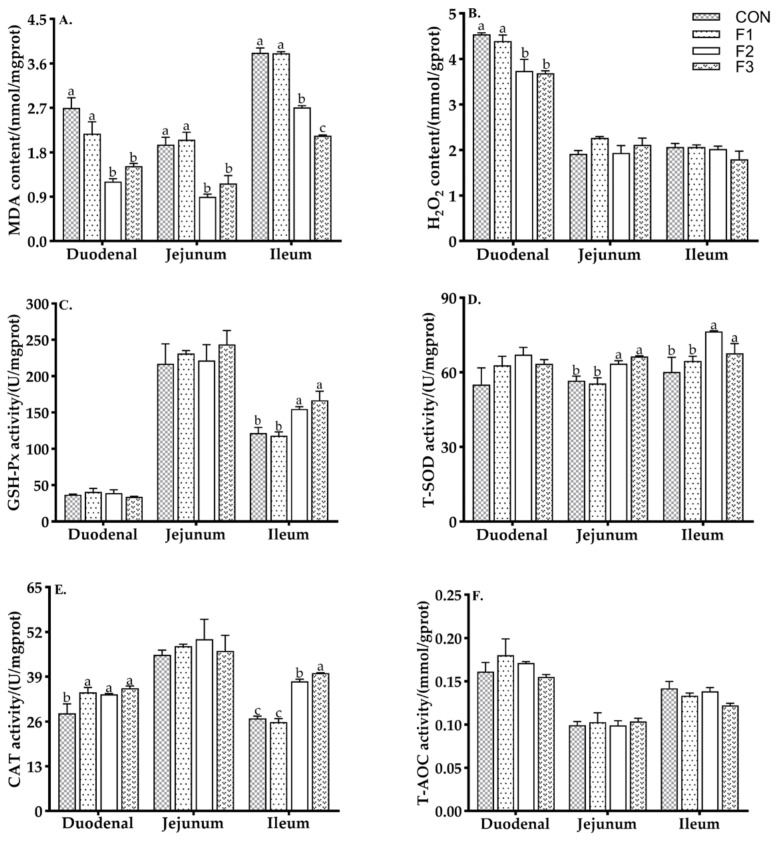
Effects of dietary fucoidan supplementation on intestinal mucosa antioxidant capacity in weaned goat kids. (**A**) MDA content; (**B**) H_2_O_2_ content; (**C**) GSH-Px activity; (**D**) T-SOD activity; (**E**) CAT activity; (**F**) T-AOC activity. Results are presented as mean ± SEM. ^a–c^ means significantly different (*p* < 0.05). CON, basal diet; F1, basal diet + 0.1% fucoidan; F2, basal diet + 0.3% fucoidan; F3, basal diet + 0.5% fucoidan. MDA, malondialdehyde; H_2_O_2_, hydrogen peroxide; GSH-Px, Glutathione peroxidase; T-SOD, total superoxide dismutase; CAT, catalase; T-AOC, total antioxidant capacity.

As shown in Figure 2, goat kids fed the F2 and F3 diets had decreased MDA content in the duodenum, jejunum, and ileum and H_2_O_2_ in the duodenum compared to goat kids fed the CON and F1 diets (*p* < 0.05). Goat kids fed diets with fucoidan had increased activity of CAT in the duodenum compared to goat kids fed the CON diet. Goat kids fed the F2 and F3 diets had increased activities of T-SOD in the jejunum and ileum and CAT in the ileum compared to goat kids fed the CON and F1 diets (*p* < 0.05).

### 3.4. Intestinal Mucosal Immunity

**Figure 3:**
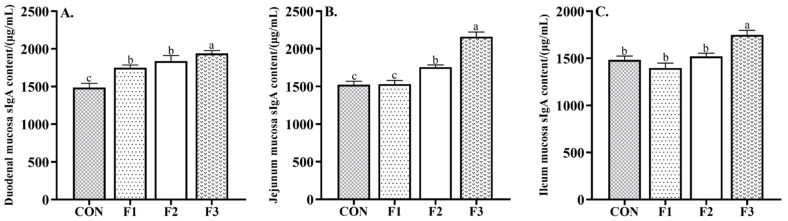
Effects of dietary fucoidan supplementation on intestinal mucosal immunity in weaned goat kids. (**A**) Duodenal mucosa sIgA content; (**B**) jejunum mucosa sIgA content; (**C**) ileum mucosa sIgA content. Results are presented as mean ± SEM. ^a–c^ means significantly different (*p* < 0.05). CON, basal diet; F1, basal diet + 0.1% fucoidan; F2, basal diet + 0.3% fucoidan; F3, basal diet + 0.5% fucoidan. sIgA, secretory immunoglobulin A.

As shown in Figure 3, goat kids fed diets with fucoidan had increased content of sIgA in the duodenal mucosa compared to goat kids fed the CON diet. Goat kids fed F2 and F3 diets had increased content of sIgA in the jejunum mucosa compared to goat kids fed the CON and F1 diets (*p* < 0.05). Goat kids fed the F3 diet had increased content of sIgA in the ileum mucosa compared to goat kids fed the CON, F1, and F2 diets (*p* < 0.05).

### 3.5. Intestinal Tight Junction Protein mRNA Expression

**Figure 4:**
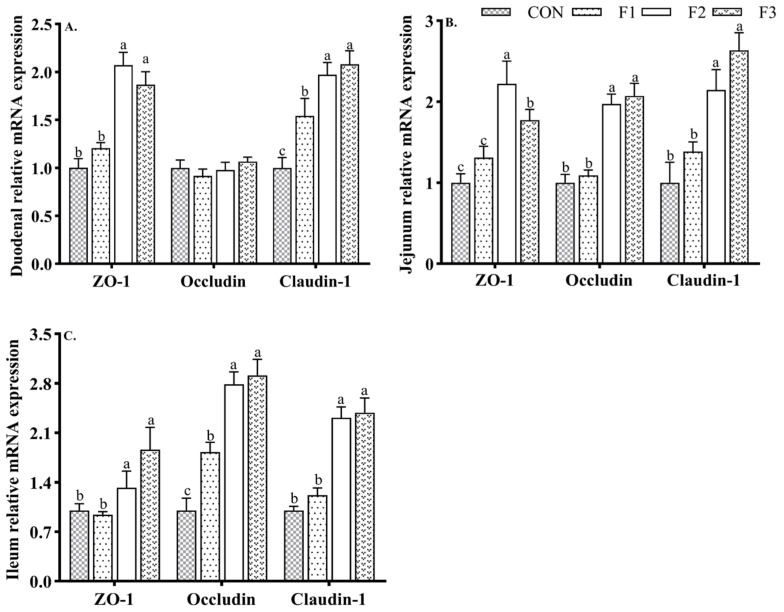
Effects of dietary fucoidan supplementation on intestinal tight junction protein mRNA expression level in weaned goat kids. (**A**) Duodenal tight junction protein mRNA expression level; (**B**) jejunum tight junction protein mRNA expression level; (**C**) ileum tight junction protein mRNA expression level. Results are presented as mean ± SEM. ^a–c^ means significantly different (*p* < 0.05). CON, basal diet; F1, basal diet + 0.1% fucoidan; F2, basal diet + 0.3% fucoidan; F3, basal diet + 0.5% fucoidan. ZO-1, zonula occludens.

As shown in Figure 4, goat kids fed diets with fucoidan upregulated the gene expression of claudin-1 in the duodenal mucosa compared to those fed the CON diet. Goat kids fed the F2 and F3 diets displayed upregulated (*p* < 0.05) the gene expression of ZO-1 in the duodenum, and the gene expression levels of ZO-1, occludin, and claudin-1 in the jejunum and ileum were higher than in goat kids fed the CON and F1 diets.

### 3.6. Alteration in the Composition of the Cecal Bacterial Community

**Figure 5:**
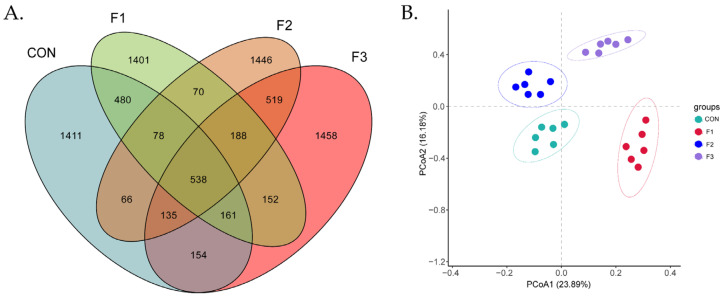
Diversity analysis. (**A**) Venn diagrams of OTUs sharing >97% nucleotide sequence identity; (**B**) principal co-ordinates (PCoA) analysis of OUTs; CON, basal diet; F1, basal diet + 0.1% fucoidan; F2, basal diet + 0.3% fucoidan; F3, basal diet + 0.5% fucoidan; OTUs, operational taxonomic units; PCA, principal component analysis.

Operational taxonomic units were identified using the criterion of 97% identity. The CON group contained 3023 OTUs; the F1 group contained 3068 OTUs; the F2 group contained 3040 OTUs, and the F3 group contained 3170 OTUs. There were 538 shared OTUs and 1141, 1401, 1446, and 1458 OTUs specific to the respective four groups. The distance between points represented the difference in the composition of the flora (Figure 5A). The principal coordinate 1 explained 23.89% of the variation among microbial colonies in all cecal contents, and the principal coordinate 2 explained 16.18% of the variation among microbial colonies in all cecal contents. The figure shows that the samples from the F1, F2, and F3 groups were clearly separated from the CON samples. Thus, fucoidan affected the cecal contents in goat kids (Figure 5B).

**Figure 6:**
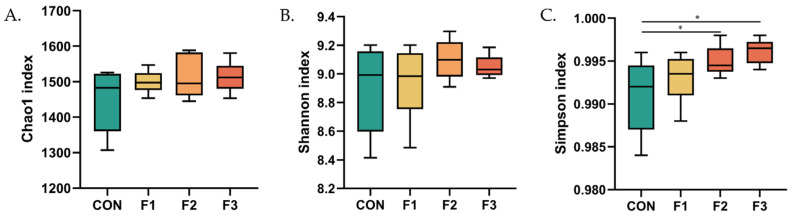
α-diversity index of gut microbiota. (**A**) Chao1 index; (**B**) Shannon index; (**C**) Simpson index. CON, basal diet; F1, basal diet + 0.1% fucoidan; F2, basal diet + 0.3% fucoidan; F3, basal diet + 0.5% fucoidan; * *p* < 0.05.

As shown in Figure 6, goat kids fed with the F2 and F3 diets had increased values of the Simpson index compared to goat kids fed the CON and F1 diets. No significant differences were observed in the values of the Chao1 or Shannon indices among treatments.

### 3.7. Alteration of Cecal Microbiota Composition Caused from Fucoidan

**Figure 7:**
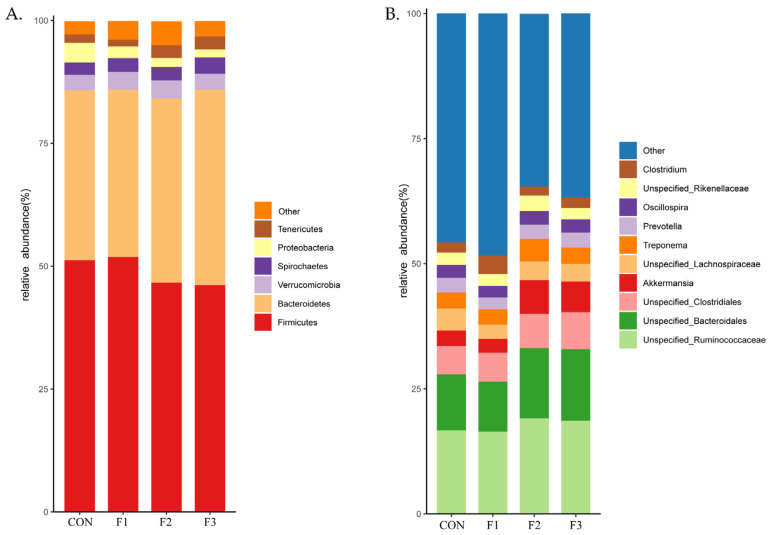
Bacterial composition at phylum (**A**) and genus (**B**) level in cecal content of goat kids among four dietary treatments. CON, basal diet; F1, basal diet + 0.1% fucoidan; F2, basal diet + 0.3% fucoidan; F3, basal diet + 0.5% fucoidan.

At the phylum level (Figure 7A), species annotation results showed that the abundances of *Firmicutes* and *Bacteroidetes* accounted for more than 70% of the cecal microbiota composition, and these were the dominant phyla in the cecal microbiota of goat kids. At the genus level (Figure 7B), species annotation results showed that the abundance of *Unspecified_Ruminococcaceae*, *Unspecified_Bacteroidale,* and *Unspecified_Clostridiales* accounted for more than 30% of the genera in the cecal microbiota in weaned goat kids.

**Figure 8:**
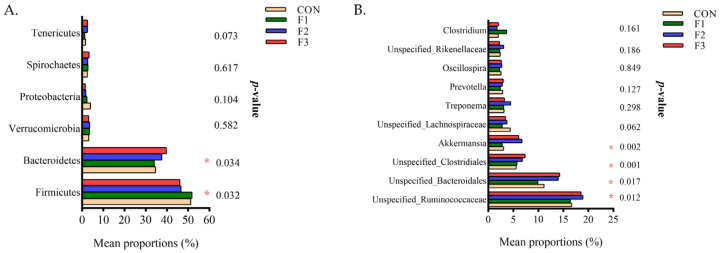
Analysis for different bacterial at phylum (**A**) and genus (**B**) level among four dietary treatments. CON, basal diet; F1, basal diet + 0.1% fucoidan; F2, basal diet + 0.3% fucoidan; F3, basal diet + 0.5% fucoidan. * *p* < 0.05.

At the phylum level (Figure 8A), compared to the CON group, goat kids fed diets with fucoidan supplementation showed increased (*p* < 0.05) the abundance of *Bacteroidetes* and decreased (*p* < 0.05) the abundance of *Firmicutes*. At the genus level (Figure 8B), compared with the CON group, goat kids fed the F2 and F3 diets had increased abundances of *Unspecified_Ruminococcaceae*, *Unspecified_Bacteroidale, Unspecified_Clostridiales*, and *Akkermansia* (*p* < 0.05).

## 4. Discussion

### 4.1. Serum Biochemical Indices

Serum biochemical parameters are important indices that reflect the metabolism and health status of goat kids [28]. In this study, biochemical parameters and enzyme activities in the serum were within the reference value ranges established for goat kids [29]. LDH is a key enzyme that catalyzes pyruvate to lactate and has been demonstrated to be upregulated in hepatitis and kidney disease [30]. In our study, dietary fucoidan reduced the activity of LDH. Similarly, Piner et al. [31] reported that the administration of 50 mg/kg fucoidan for 7 days decreased the activity of LDH and exhibited a protective role as an antioxidant against sulfoxaflor-induced oxidative stress. In addition, transaminases are enzymes that catalyze the transformation of amino acids to keto acids, and ALT and AST are two of the most important transaminases [32]. The activity of AST in serum is increased in liver damage. In this study, dietary fucoidan supplementation reduced the activity of AST as measured on day 30. In accordance with our study, Choi et al. [33] reported that dietary supplementation with 0.02 g/kg fucoidan improved the aspirin-induced stomach ulceration in rats and reduced the AST activity. The contents of TP, GLU, TG, and T-CHO in serum indicate the metabolic status of proteins, sugars, and fats in ruminants [34,35]. Our results have shown that dietary fucoidan administration reduced the content of GLU but increased the content of TP. Similarly, Abdel et al. [36] reported that dietary fucoidan protected *Nile tilapia* from the toxicity of waterborne atrazine and increased the content of TP in the serum. In summary, dietary fucoidan does not adversely affect the liver or kidneys of goat kids. According to our previous results [23], we speculate that dietary fucoidan supplementation increases the utilization of TP, thus promoting the growth of goat kids.

### 4.2. Small Intestinal Barrier Function

The small intestine is not only an important site for digestion and absorption but also a barrier to pathogens and toxins [37]. The maintenance of gut integrity is necessary for normal functioning. Previous research has shown that the effects of weaning on the intestinal barrier function of goat kids may be due to stress [38]. D-lactic acid (D-LA) is an indicator of bacterial metabolites and bacterial lysis. The content of D-LA increases in serum with increased intestinal mucosal permeability [39]. DAO is a highly active intracellular enzyme that is a reliable serum marker for the indirect assessment of the mucosal epithelial cell layer [40]. In this study, dietary fucoidan administration decreased the content of D-LA and the activity of DAO in the serum of goat kids. In accordance with our study, Xue et al. [41] indicated that fucoidan reduced the content of D-LA and the activity of DAO in the serum of rats and alleviated intestinal barrier injury caused by breast cancer. Weaning led to the metabolic disorders of oxygen-free radicals, resulting in oxidative stress [42]. The SOD, GSH-Px, and CAT enzymes are the first line of defense against oxidative injury, and MDA and H_2_O_2_ are the final products of lipid peroxidation [42,43]. In this study, dietary fucoidan administration decreased the contents of MDA and H_2_O_2_ and increased the activities of GSH-Px, SOD, and CAT, resulting in increased intestinal antioxidant function. Similarly, Li et al. [44] reported that fucoidan improved the oxidative damage to the porcine intestinal epithelial cells by increasing the activities of GSX and GSH and decreasing the content of MDA. The protein sIgA is the most abundant immunoglobulin and the principal regulator of adaptive defenses on mucosal surfaces [45]. In our study, fucoidan increased the contents of sIgA of the duodenum, jejunum, and ileum mucosa in weaned goat kids. Tight junctions are the structural barriers that prevent antigens from moving from the intestinal lumen to the body tissues via the paracellular pathway [46]. Tight junctions are composed of a complex of proteins including claudin, occludin, and zonula occludens. Research has confirmed that weaning stress resulted in damage to the intestinal barrier in goat kids and reduced the mRNA expression of tight junction proteins in the small intestine [47]. In this study, dietary fucoidan supplementation increased the mRNA expression levels of ZO-1, *claudin-1*, and *occludin* in the duodenum, jejunum, and ileum. Our results were in line with those of Iraha et al. [48], who reported that fucoidan enhanced the intestinal barrier function by upregulating the expression of *Claudin-1*. Taken together, the results suggest that improvement of intestinal barrier function by dietary fucoidan may be related to increases in the physical barrier, immunological barrier, and mucosal antioxidant capacity.

### 4.3. Cecal Microbial Community

The gut microbiome in ruminants has been shown to play an important role in the extraction of valuable nutrients from feed and thereby preserving host health [49]. The richness, diversity, and evenness of microbial communities of samples can be suggested by OTUs and alpha-diversity analysis. In our study, the samples from the fucoidan treatment groups had higher numbers of OTUs and higher values of the Simpson index than samples from the CON group. These results suggest that dietary fucoidan supplementation may increase the diversity of the cecum microflora of weaned goat kids. In addition, we explored the impact of fucoidan on the composition of the cecal bacterial community. Previous studies have suggested that the dominant microflora in the ruminant gut at the phylum level were *Bacteroidetes* and *Firmicutes* and that the relative abundances could vary [50]. Consistent with previous studies, we found that the phyla *Firmicutes* and *Bacteroidetes* dominated the cecal core microbiome in the goat kids. Previous research has shown that *Firmicutes* play a major role in fiber degradation and that *Bacteroidetes* are involved in fermenting carbohydrates [51]. However, *Proteobacteria* includes a variety of opportunistic pathogens, and metabolic disorders and the intestinal inflammatory response may be due to the increased abundance of *Proteobacteria* [52]. Our study found that fucoidan increased the abundance of the phyla *Bacteroidetes*, *Tenericutes*, and *Fibrobacteres* and decreased the abundances of the phyla *Firmicutes*, *Proteobacteria*, and *TM7* in the cecum. At the genus level, *Unspecified_Ruminococcaceae, Unspecified_Bacteroidales,* and *Unspecified_Clostridiales* were the dominant bacterial genera in the cecum of weaned goat kids, consistent with the previous findings by Gu et al. [53]. Previous research has shown that some genera in *Ruminococcaceae* could produce cellulase and amylase [54]. The genus *Clostridium* are beneficial bacteria commonly found in the intestine that ferment fibrous material to produce butyric acid in stimulating the mucosal immune response [55]. In our study, dietary supplementation with fucoidan increased the abundances of *Unspecified_Ruminococcaceae, Unspecified_Bacteroidales, Unspecified_Clostridiales*, and *Akkermansia* in the cecum. These results suggest that active carbohydrate fermentation may be present in the cecum of goat kids and that fucoidan played a regulatory role in the intestinal microflora of the goats.

## 5. Conclusions

In this study, it was found that dietary fucoidan supplementation improved the small intestinal barrier function and cecal microflora balance in weaned goat kids. This suggested that the supplements could be used to promote the gut health of juvenile goats.

## Figures and Tables

**Table 1:** The composition and level of basal diet (air-dry basis).

Items	Content
Ingredients (%)	
Pennisetum purpureum	35.00
Corn	40.89
Soybean meal	13.98
Wheat bran	7.15
Salt	0.65
CaHPO_4_	0.84
Limestone	0.84
Premix ^1^	0.65
Total	100
Nutrient level	
DM (%)	88.86
ME (MJ/kg)	10.43
CP (%)	12.06
NDF (%)	30.06
ADF (%)	16.39
Ca (%)	0.76
*p* (%)	0.54

^1^ The premix provided the following per kg of diets: VA 8 000 IU, VD 2 000 IU, VE 40 IU, Cu 12 mg, Fe 70 mg, Mn 50 mg, Zn 80 mg, I 1.0 mg, Se 0.27 mg, and Co 0.3 mg. CaHPO_4_, calcium hydrogen phosphate; DM, dry matter; ME, metabolizable energy; CP, crude protein; NDF, neutral detergent fiber; ADF, acid detergent fiber; Ca, calcium; P, phosphorus.

**Table 2:** Nucleotide sequences of primers used to measure targeted genes.

Gene Symbols	Product Length, bp	Accession No.	Nucleotide Sequence of Primers (5′→3′)
ZO-1	111	XM_018066118.1	F: CGTCCTGATCCTGAACCTGTGTCT
			R: GCTCTTCTCGCTCCTCCTGTGT
Occludin	157	XM_018065681.1	F: CCTGTGTTGCCTCCACTCTT
			R: TCCGTATAGCCTGTTCCATAGC
Claudin-1	95	XM_005675123.3	F: CCTGCTGGGACTAATAGCCA
			R: CAGCCATTCGCATCTTCTGT
β-actin	91	NM_001314342.1	F: TCCTTCCTGGGCATGGAATC
			R: CGTAAAGGTCCTTGCGGATG

ZO-1, zonula occludens.

**Table 3:** Effects of dietary fucoidan on serum biochemical in weaned goat kids.

Item ^1^	CON	F1	F2	F3	SEM ^2^	*p*-Value
15 d						
ALT (U/L)	2.87	2.17	2.90	3.30	0.66	0.40
AST (U/L)	13.97	13.24	11.42	9.76	2.90	0.48
LDH (U/L)	4359.98 ^a^	3434.89 ^b^	3505.28 ^b^	3656.11 ^b^	371.55	0.03
TG (mmol/L)	1.00	0.93	0.80	0.97	0.12	0.37
T-CHO (mmol/L)	1.92	1.32	1.57	1.77	0.22	0.05
GLU (mmol/L)	0.73 ^a^	0.69 ^b^	0.60 ^c^	0.62 ^c^	0.04	0.01
TP (mg/mL)	27.69 ^b^	30.15 ^b^	30.40 ^b^	35.84 ^a^	2.00	0.01
30 d						
ALT (U/L)	2.74	4.19	3.38	3.05	0.75	0.26
AST (U/L)	18.78 ^a^	11.85 ^b^	10.34 ^b^	9.10 ^b^	2.55	0.02
LDH (U/L)	5174.24 ^a^	5851.01 ^a^	4848.49 ^b^	4093.43 ^b^	584.36	0.04
TG (mmol/L)	0.81	0.98	0.97	0.68	0.14	0.14
T-CHO (mmol/L)	1.49	1.38	1.64	1.76	0.15	0.09
GLU (mmol/L)	0.67	0.64	0.64	0.57	0.38	0.10
TP (mg/mL)	88.49 ^c^	107.08 ^b^	110.92 ^a^	119.06 ^a^	5.11	<0.01

^1^ CON, basal diet; F1, basal diet + 0.1% fucoidan; F2, basal diet + 0.3% fucoidan; F3, basal diet + 0.5% fucoidan. ALT, alanine aminotransferase; AST, aspartate aminotransferase; LDH, lactate dehydrogenase; TG, total glyceride; T-CHO, total cholesterol; GLU, glucose; TP, total protein. ^2^ SEM, standard error of mean. ^a–c^ Values in the same row with different letters are significant different (*p* < 0.05). Results are presented as mean ± SEM.

## Data Availability

The data presented in this study were available on request from the corresponding author.
